# Carbon monoxide alleviates senescence in diabetic nephropathy by improving autophagy

**DOI:** 10.1111/cpr.13052

**Published:** 2021-05-07

**Authors:** Li Chen, Guibin Mei, Chunjie Jiang, Xueer Cheng, Dan Li, Ying Zhao, Huimin Chen, Cheng Wan, Ping Yao, Chao Gao, Yuhan Tang

**Affiliations:** ^1^ Hubei Key Laboratory of Food Nutrition and Safety Ministry of Education Key Laboratory of Environment and Health and MOE Key Laboratory of Environment and Health Key Laboratory of Environment and Health (Wuhan) Ministry of Environmental Protection State Key Laboratory of Environment Health (Incubation) Department of Nutrition and Food Hygiene School of Public Health Tongji Medical College Huazhong University of Science and Technology Wuhan China; ^2^ Department of Nephrology Union Hospital Tongji Medical College Huazhong University of Science and Technology Wuhan China; ^3^ Key Laboratory of Trace Element Nutrition of National Health Commission Chinese Center for Disease Control and Prevention National Institute for Nutrition and Health Beijing China

**Keywords:** autophagy, carbon monoxide, diabetic nephropathy, senescence

## Abstract

**Objectives:**

Senescence, characterized by permanent cycle arrest, plays an important role in diabetic nephropathy (DN). However, the mechanism of renal senescence is still unclear, and the treatment targeting it remains to be further explored.

**Materials and Methods:**

The DN mice were induced by HFD and STZ, and 3 types of renal cells were treated with high glucose (HG) to establish in vitro model. Senescence‐related and autophagy‐related markers were detected by qRT‐PCR and Western blot. Further, autophagy inhibitors and co‐immunoprecipitation were used to clarify the mechanism of CO. Additionally, the specific relationship between autophagy and senescence was explored by immunofluorescence triple co‐localization and ELISA.

**Results:**

We unravelled that senescence occurred in vivo and in vitro, which could be reversed by CO. Mechanistically, we demonstrated that CO inhibited the dysfunction of autophagy in DN mice partly through dissociating Beclin‐1‐Bcl‐2 complex. Further results showed that autophagy inhibitors blocked the improvement of CO on senescence. In addition, the data revealed that autophagy regulated the degradation of senescence‐related secretory phenotype (SASP) including *Il‐1β*, *Il‐6*, *Tgf‐β* and *Vegf*.

**Conclusions:**

These results suggested that CO protects DN mice from renal senescence and function loss via improving autophagy partly mediated by dissociating Beclin‐1‐Bcl‐2 complex, which is possibly ascribed to the degradation of SASP. These findings bring new ideas for the prevention and treatment of DN and the regulation of senescence.

## Graphical abstract

In diabetic nephropathy, senescent cells were accumulated, which may accelerate disease progression through senescence‐related secretory phenotype (SASP). Carbon monoxide activated autophagy by dissociating Beclin‐1‐Bcl‐2 complexe, and restore lysosomal function to improve autophagy flow, enabling autophagosomes to encapsulate some SASP and deliver it to lysosome for degradation. Thus, the reversion of SASP from secretion to degradation prevented senescence and subsequently improved DN.

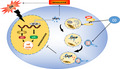

## 1 INTRODUCTION

Diabetic nephropathy (DN) is one of the most common and serious diabetic microvascular complications. Statistically, 20%‐40% of patients with DN will develop into end‐stage renal disease (ESRD) at a rate of 14 times faster than other renal diseases.^1^ Additionally, the increased cardiovascular risk of DN patients greatly contributes to high mortality.^2^ The complex pathogenesis of DN is still unclear with limited treatment approach available. Hence, it is urgent to identify the pathogenesis and therapeutic agents to prevent the progressive loss of renal function of DN.

Senescent cells, characterized by permanent cell cycle arrest, have attracted extensive attention as ‘zombie cells’. Due to resistance to apoptosis and the continued production of senescence‐associated secretory phenotype (SASP), senescent cells are increasingly recognized as the crucial cause for age‐related diseases.^3^ Senescence linked renal dysfunction has been commonly observed in DN patients and experimental models, such as STZ induced (type 1 diabetes (T1D)) and db/db (type 2 diabetes (T2D) ) mice.^4^, ^5^ Importantly, the clearance of p16‐positive cells of aged mice improved glomerulosclerosis.^6^ Furthermore, knocking out p21 or p27 in T1D mice relieved proteinuria and glomerular dilation.^7^, ^8^ These results suggested that senescence plays an important role in the pathogenesis of DN. However, the regulation of senescence in DN remains vague.

Emerging evidence showed that autophagy, a mechanism of the degradation of intracellular contents, is a valid senescence regulator.^9^ Although it has been reported that autophagy promotes the acquisition of senescent phenotype, the anti‐senescence effect of autophagy is more widely accepted. Upregulation of autophagy was reported to extend the lifespan of aged mice and elder flies.^10^ Further studies showed that knocking out Atg7 aggravated stem cell senescence.^11^ More importantly, the deletion of Atg5 aggravated proteinuria and fibrosis, the main performance of kidney ageing.^12^, ^13^ Thus, drugs targeting autophagy to alleviate senescence may be one of the most promising strategies for DN prevention and treatment.

Carbon monoxide (CO) generated via the catabolism of haem by haem oxygenase enzymes is an endogenously gaseous molecule. A number of publications revealed the anti‐inflammatory, anti‐apoptotic and other protective properties of CO when applied at low doses.^14^ Although CO has been well‐studied to confer renoprotection to ischaemia‐reperfusion or kidney transplantation mice,^15^ the effect and mechanism of CO on DN are unclear. Recently, literature showed that CO imparted cytoprotective roles as an autophagy activator in islets challenged by hypoxia,^16^ sepsis mice^17^ and aged rats with cardiac arrest.^18^ Moreover, the administration of CO alleviated the senescence of endothelial cells caused by drug toxicity.^19^ These findings apparently advance the potential mediation of autophagy targeted by CO to ameliorate senescence.

Hence, we hypothesized that the accumulation of senescent cells in the kidney of DN mice could be reversed by CO via autophagy activation, subsequently improving renal dysfunction. To test our hypothesis and explore the underlying mechanisms, we applied the treatment of carbon monoxide releasing molecule‐2 (CORM‐2) in vivo (the experimental DN mice) and in vitro (rat mesangial cells, human tubular epithelial cells and human podocyte).

## 2 MATERIALS AND METHODS

### 2.1 Animal experimental design

Eight‐week‐old male C57BL/6J mice (Charles River) were kept in a standard light/dark cycle (12:12 hours) with normal diet (ND) or high‐fat diet (HFD)(60% energy from fat) for 16 weeks.^20^ At week 17 of the study, mice fed with HFD received intraperitoneal injections of STZ (50 mg/kg) for 7 days, while mice with ND were injected with vehicle (citrate buffer, pH = 4.5). One week after the injections, blood glucose levels of a tail prick were measured twice at a 24 hours interval. Next, the DN mice with glucose levels of 16.9 mmol/L or greater were divided into three groups randomly, with 15 animals in each group (DN, DN + CO, and DN + iCO): a group termed as DN control and other groups treated with intraperitoneal injections (twice a week for 16 weeks) of CORM‐2 (3 mg/kg) or invalid CORM‐2 which was produced by releasing CO from CORM‐2 at room temperature. At the end of the experimental period, mice were anaesthetized, blood samples were collected by enucleation of eyeballs, and kidneys were harvested for analysis. All animals were treated in accordance with the Guiding Principles in the Care and Use of Laboratory Animals published by the US National Institutes of Health, and all animal procedures were approved by the Tongji Medical College Council on Animals Care Committee.

### 2.2 Plasma biochemical and histological analysis

The collected whole blood and serum was stored at −80℃ for analysis. Blood urea nitrogen (BUN) was measured with commercially available assay kits (Jiancheng Bioengineering Institute, China). After kidneys were removed and the surface was washed with saline. The kidney tissue was stained with haematoxylin‐eosin and Masson's trichrome stain and finally observed with a microscope.

### 2.3 Electron microscopy

Small pieces of renal cortex were fixed in glutaraldehyde (2.5%) and embedded in araldite. The tissue was polymerized cut into ultrathin sections (80‐100 nm) using an ultramicrotome (Leica EM UC7). The thin slices on copper mesh grid were stained and observed under a transmission electron microscope (Tecnai G220 TWIN).

### 2.4 Shear wave elastography

At the end of the experimental period, the mice were anaesthetized and sent to the ultrasonic laboratory (Union Hospital, Tongji Medical College, Huazhong University of Science and Technology, Wuhan, China) for shear wave elastography using a sonoscope (Supersonic Imagine). The mean Young's modulus (Emean) was used to measure renal parenchymal stiffness.

### 2.5 SA‐β‐gal staining

Frozen kidney sections and cells seeded in a 6‐well plate were used for detection of SA‐β‐gal activity by a commercial kit (Beyotime Biotechnology) according to the manufacturer's instructions. The blue stain was considered as the accumulation areas of senescent cells.

### 2.6 Real‐time quantitative PCR for mRNA expression

According to the instructions (TaKaRa BIO INC), renal tissue RNA was extracted using the TRIzol reagent. The expression of mRNA was quantified with TB green‐based qRT‐PCR kit and specific primers (Table 1). Each gene expression was assessed with its own standard curve, and the mRNA level of *Gapdh* was quantified as an endogenous control.

**TABLE 1:** Real‐time quantitative PCR primer sequences

Gene name	primer sequence(5'‐3')
*Gapdh*	CCTCGTCCCGTAGACAAAATG
	TGAGGTCAATGAAGGGGTCGT
*Il‐6*	TTCTTGGGACTGATGCTGGTG
	GCCATTGCACAACTCTTTTCTC
*Il‐1β*	GCATCCAGCTTCAAATCTCGC
	TGTTCATCTCGGAGCCTGTAGTG
*Tnf‐α*	CCCTCACACTCACAAACCACC
	CTTTGAGATCCATGCCGTTG
*Vegf*	CACTGGACCCTGGCTTTACTG
	CTCAATCGGACGGCAGTAGC
*Icam‐1*	CTCGGAAGGGAGCCAAGTAAC
	CAGCCGAGGACCATACAGCA
*Vcam‐1*	AGATAGACAGCCCACTAAACGC
	CAGCCTGTAAACTGGGTAAATGT
*Tgf‐β*	CAACAATTCCTGGCGTTACCT
	GCCCTGTATTCCGTCTCCTT

### 2.7 Western blotting and immunoprecipitation

Renal tissue or cell was homogenized and lysed, then quantified by BCA protein assay kit (Beyotime Biotechnology). The protein was separated and subsequently transferred onto the PVDF membranes (Millipore). After being blocked, the membranes were incubated overnight with primary antibodies (Table 2). After washing, the membranes were incubated with corresponding secondary antibody. The density of each target band was quantified by Image Pro‐Plus 6.0 software and normalized to GAPDH as optical density. All sample sizes of animals or cells were greater than or equal to 3.

**TABLE 2:** All antibody lists

Antibodies	Host	Dilution	Company and location
p53	Mouse monoclonal	1:1000 for WB	Cell Signaling Technology, USA
p21	Rabbit monoclonal	1:1000 for WB	Abcam, USA
p21	Rabbit polyclonal	1:1000 for WB	Proteintech, China
p16	Rabbit monoclonal	1:1000 for WB	Abcam, USA
p15/p16	Mouse monoclonal	1:200 for WB	Santa Cruz, USA
LC3B	Rabbit monoclonal	1:1000 for WB 1:100 for IF	Cell Signaling Technology, USA
MAP‐LC3β	Mouse monoclonal	1:100 for IF	Cell Signaling Technology, USA
p62	Rabbit polyclonal	1:1000 for WB	Proteintech, China
Beclin‐1	Rabbit monoclonal	1:1000 for WB	Cell Signaling Technology, USA
Atg7	Rabbit polyclonal	1:1000 for WB	ABclonal, China
LAMP2	Mouse monoclonal	1:1000 for WB 1:100 for IF	Proteintech, China
LAMP2	Rat monoclonal	1:50 for IF	Santa Cruz, USA
Cathepsin B	Rabbit polyclonal	1:200 for WB	Santa Cruz, USA
GATA4	Rabbit polyclonal	1:1000 for WB	Proteintech, China
NF‐kappaB p65	Rabbit monoclonal	1:100 for IF	Cell Signaling Technology, USA
mTOR	Rabbit monoclonal	1:100 for IF	Cell Signaling Technology, USA
IL‐1β	Rabbit polyclonal	1:50 for IF	ABclonal, China
IL‐6	Rabbit polyclonal	1:50 for IF	ABclonal, China
TGF‐β	Rabbit monoclonal	1:50 for IF	Santa Cruz, USA
VEGF	Rabbit polyclonal	1:50 for IF	Proteintech, China
Bcl‐2	Mouse monoclonal	1:1000 for WB	Proteintech, China
GAPDH	Mouse monoclonal	1:5000 for WB	Proteintech, China
GAPDH	Rabbit monoclonal	1:5000 for WB	Proteintech, China
LysoTracker Red			Thermo Scientific, USA
Alexa fluor 488‐conjugated goat anti‐rabbit IgG		1:800 for IF	Thermo Scientific, USA
Alexa fluor 555‐conjugated goat anti‐mouse IgG		1:800 for IF	Thermo Scientific, USA
Alexa fluor 647‐conjugated goat anti‐rat IgG		1:800 for IF	Thermo Scientific, USA

Lysate of kidney tissue was centrifuged (14 000 *g* at 4°C for 30 minutes), and the supernatant was pre‐cleared with 20 μL protein A/G agarose beads for 2 hours and incubated overnight with Bcl‐2 or mouse IgG antibody. Immunoprecipitates were washed, resuspended, boiled and analysed by Western blot using anti‐Beclin‐1 (Cell Signaling Technology) and anti‐Bcl‐2 (Proteintech) as described earlier.^21^

### 2.8 Immunofluorescence

The renal tissue embedded in OCT media was cut into 6‐8 μm thick frozen sections. After blocked with 10% normal goat serum, the sections incubated overnight with primary antibodies. The following day, the tissue was labelled with secondary antibodies. After rinsed with PBS, the slices were incubated with DAPI and subsequently photographed under a fluorescent microscope (Olympus).

### 2.9 Cell culture

HBZY‐1 were maintained in DMEM (Gibco) and HK‐2 were maintained in DMEM/F12 (Gibco) supplemented with 10% foetal bovine serum, 100 U mL^–1^ penicillin/streptomycin (Gibco) at 37°C in a humidified atmosphere containing 95% air and 5% CO_2_. HPC provided by Professor Chun Zhang from Huazhong University of Science and Technology were maintained in RPMI 1640 (Gibco). HPC was first proliferated at 33℃ and then transferred to 37℃ for differentiation before it can be used in experiments as described earlier.^22^

### 2.10 ELISA assay

After HPC was treated differently and cultured for 5 days, half of the supernatant was taken out for detection (released VEGF). The cells were broken using an ultrasonic cell crusher (SONICS), allowing the proteins to be released completely into the remaining supernatant (total VEGF). Then, the VEGF concentrations were measured using a commercial ELISA kit (MEIMIAN). The ratio of released VEGF to total VEGF was the VEGF leakage rate.

### 2.11 Gene silencing

Knockdown of Atg7 in HK‐2 cells was achieved by using a reverse siRNA transfection procedure performed in six‐well plates. Once grown to 70% confluence, cells were transfected with siRNA or scrambled siRNA (RiboBio) using Lipofectamine^®^ RNAiMAX (Invitrogen) according to the manufacturer's protocol. After 48 hours of transfection, the transfection efficiency was verified by Western blot to validate the sequence (ACTCGAGTCTTTCAAGACT).

### 2.12 Monitor the autophagic flux

The plasmids containing lentiviral vector RFP‐GFP‐LC3 (Genecopoeia) were constructed and then packaged in HEK293T cells according to the manufacturer's instructions (GeneCopoeia). The virus supernatant was collected to infect HK‐2 and HBZY‐1 cells to monitor autophagy flow. This probe could distinguish autophagosomes (GFP^+^/RFP^+^, yellow puncta) and autolysosomes (GFP^−^/RFP^+^, red puncta).

### 2.13 Data analysis

The data were analysed using Graph Pad Prism 8 and showed as the mean ± SEM. Differences among the groups were determined by one‐way analysis of variance. Significance was set as *P* < .05.

## 3 RESULTS

### 3.1 CO attenuated renal dysfunction of DN mice

**FIGURE 1:**
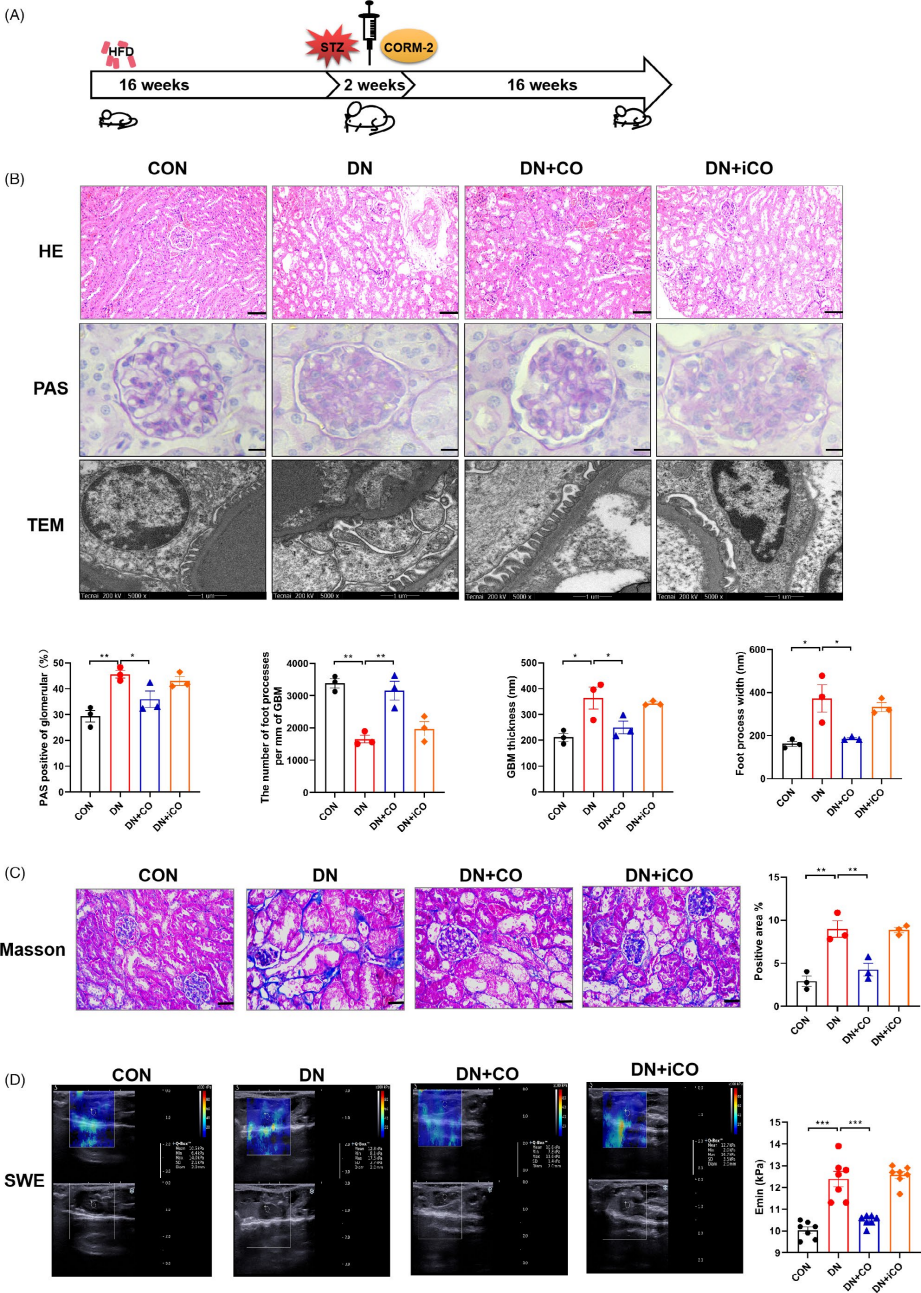
CO attenuated renal dysfunction of DN mice induced by HFD and STZ. A, Schema of experimental design. B, Representative images of kidney tissue stained with H&E (scale bar: 50 μm), PAS (scale bar: 10 μm) and ultra‐structural changes in glomerular morphology assessed by transmission electron microscopy (scale bar: 1 μm). And the statistical data of the ratio of PAS positive to glomerular area, GBM thickness, foot process width and the number of foot processes per mm of GBM (n = 3). C, Representative images of the renal tissue stained with Masson's trichrome and the statistical data of positive area (n = 3). Scale bar: 25 μm. D, Shear wave elastography detected with a sonoscope and quantification of Young's modulus as the degree of renal fibrosis (n = 7). *P < .05, **P < .01, ***P < .001

To explore the renoprotection of CO, a DN model was induced by HFD and STZ (Figure 1A). After 34 weeks of experimental feeding, blood glucose, weight, the kidney‐body ratio, creatinine and BUN were measured. In the DN group, kidney‐body ratio, creatinine and BUN were increased, whereas the treatment of CO reversed abnormalities (Table 3). Consistently, the renal morphology of DN mice was extremely disordered with epithelial cells desquamation, vacuolar degeneration, glomerular Bowman's space enlargement and mesangial expansion (Figure 1B). Furthermore, the ultrastructure of glomerulus displayed basement membrane thickening and podocyte foot process effacement (Figure 1B). On the contrary, CO treatment normalized above negative morphological changes (Figure 1B). In addition, fibrillar collagen deposited in renal cortex (Figure 1C) and renal hardness, measured by shear wave elastography, were both increased in DN mice (Figure 1D). As expected, CO significantly improved renal dysfunction of DN including fibrosis.

**TABLE 3:** Metabolic data of mice in all groups

	CON	DN	DN + CO	DN + iCO
Plasma glucose (mmol/L)	5.74 ± 1.27	21.37 ± 1.90∗	21.29 ± 4.87	21.61 ± 4.67
Initial weight (g)	21.54 ± 0.52	21.66 ± 0.86	21.72 ± 0.72	21.12 ± 0.62
Weight at 16 wk (g)	28.18 ± 1.26	34.84 ± 1.68∗	34.73 ± 2.07	33.77 ± 2.14
Final weight (g)	27.70 ± 1.09	27.97 ± 1.46	27.83 ± 0.77	27.61 ± 1.40
Kidney weight (g)	0.31 ± 0.02	0.46 ± 0.05∗	0.36 ± 0.03^†^	0.46 ± 0.04
Kidney/body weight (%)	1.14 ± 0.06	1.65 ± 0.14∗	1.30 ± 0.10^†^	1.67 ± 0.18
Serum creatinine (μmol/L)	35.34 ± 11.81	74.06 ± 13.30∗	51.71 ± 15.17^†^	65.04 ± 14.70
Blood urea nitrogen (mmol/L)	10.16 ± 1.94	19.88 ± 1.52∗	14.53 ± 0.79^†^	18.17 ± 0.61

Note

Data were shown as mean ± SD, n = 8.

∗ *P* < .05, DN compared with CON.

^†^ *P* < .05, compared with DN.

### 3.2 CO alleviated senescence in DN mice and renal cells challenged by high glucose

**FIGURE 2:**
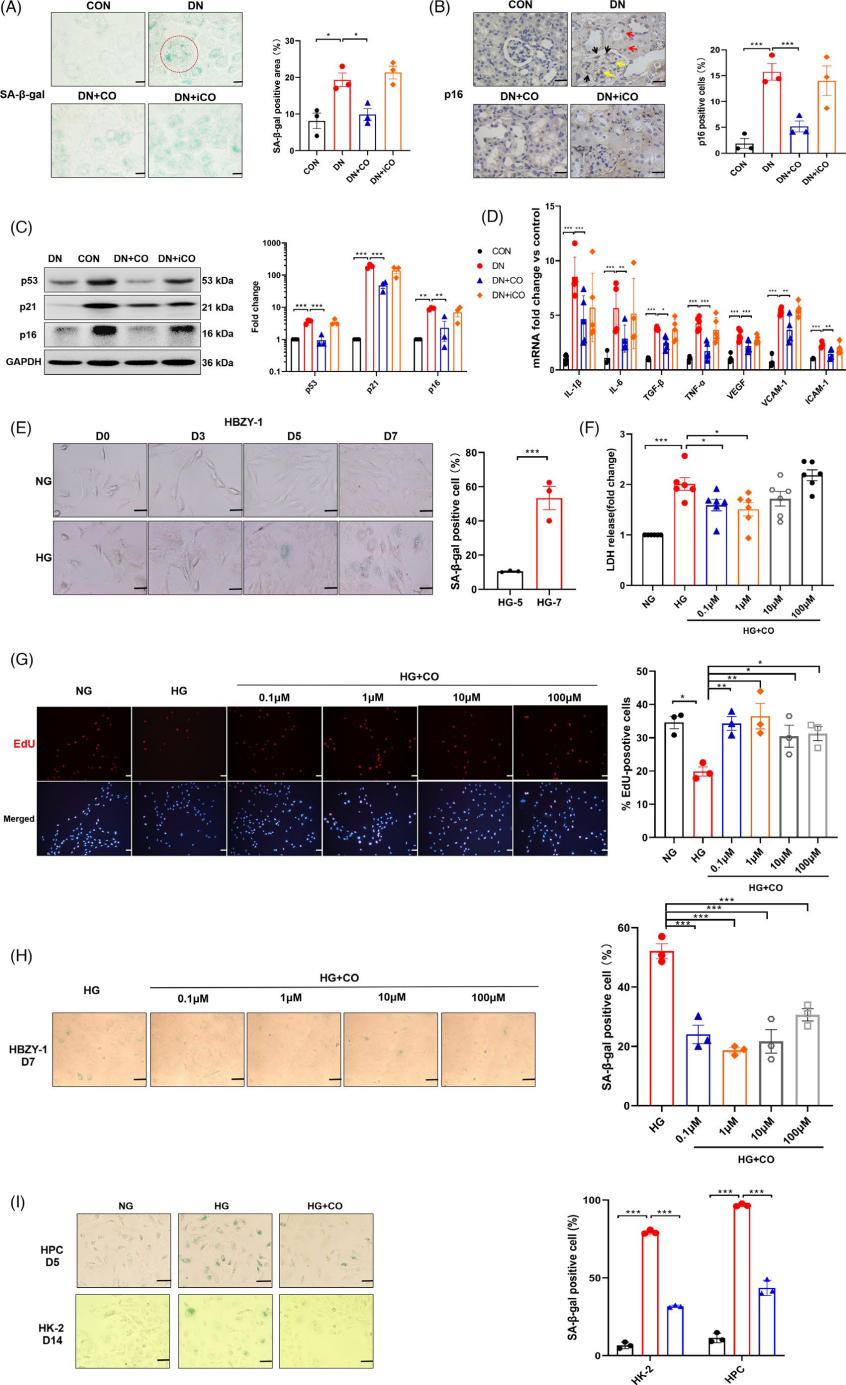
CO alleviated senescence in DN mice and renal cells challenged by HG. A, Representative images of kidney tissue stained with SA‐β‐gal and the statistical data of positive area (n = 3). The glomerulus was indicated by the red dotted circle. Scale bar: 25 μm. B, The positive expression of p16 in the kidney was shown by immunohistochemistry (n = 3). The yellow arrows indicated mesangial cells, red indicated renal tubular epithelial cells and black arrow indicated podocytes. Scale bar: 25 μm. C, The protein levels of senescence markers determined by immunoblots and densitometric analysis of p53, p21 and p16 (n = 3). D, Fold changes in expression levels of SASP mRNA (*Il‐1β, Il‐6, Tgf‐β, Tnf‐α, Vegf, Icam‐1* and *Vcam‐1*) (n ≥ 4). E, HBZY‐1 exposed to NG (5 mmol/L) or HG (35 mmol/L) was stained with SA‐β‐gal from day 0 to day 7 and statistical data of SA‐β‐gal‐positive cells at 5 and 7 d (n = 3). Scale bar: 50 μm. F, The LDH release rate of HBZY‐1 treated with different concentrations of CORM‐2 (0.1, 1, 10 and 100 μmol/L) (n = 6). G, The proportion of EdU‐positive HBZY‐1 treated with different concentrations of CORM‐2 (n = 3). H, SA‐β‐gal staining in HBZY‐1 treated with different concentrations of CORM‐2 (n = 3). Scale bar: 50 μm. I, Representative images of SA‐β‐gal of HPC (day 5) and HK‐2 (day 14) with the administration of CORM‐2 (1μmol/L) and statistical data (n = 3). Scale bar: 50 μm. *P < .05, **P < .01, ***P < .001

Senescence is increasingly considered as a main cause of renal fibrosis, a hallmark of ageing.^23^ To investigate the anti‐senescence effect of CO, we performed SA‐β‐gal staining on kidney. The increased SA‐β‐gal^+^ and p16^+^ cells were accumulated throughout the renal cortex in the DN group, while control and CO‐treatment kidneys showed occasional positivity (Figure 2A,B). From the anatomical position of kidney, the positive expression of p16 was detected in mesangial cells (yellow arrow), renal tubular epithelial cells (red arrow) and podocytes (black arrow). Senescence that occurred in DN mice was further supported by the evident upregulation of classical senescence‐associated proteins, including p53, p21 and p16 (Figure 2C). Conversely, the administration of CO alleviated the above senescent performance.

Senescence‐related secretory phenotype, the distinctive secretome of senescent cells, was reported to disrupt the microenvironment and facilitate disease progression.^24^ Hence, mRNA levels of some representative SASP were measured, including *Il‐1β, Il‐6, Tgf‐β, Tnf‐α, Vegf, Icam‐1* and *Vcam‐1*. Results showed that the abnormal increases of these SASP were averted by CO intervention (Figure 2D).

Given the suppressive senescence of CO in vivo, we further explore its anti‐senescence effect in vitro model of 3 types of renal cells, including rat mesangial cells (HBZY‐1), human tubular epithelial cells (HK‐2) and human podocyte (HPC). After exposure to high glucose (HG, 35 mmol/L), 3 types of cells displayed the augmentation of SA‐β‐gal with larger morphology at day 7 (Figure 2E), day 5 and day 14 (Figure 2I), respectively. Notably, the increase in LDH leakage rate of HBZY‐1 indicated that HG induced cell senescence and damage (Figure 2F). In order to investigate the appropriate dose of CO, HBZY‐1 was treated with different concentrations of CORM‐2. By measuring leakage rate of LDH (Figure 2F), EdU‐positive cells (Figure 2G) and SA‐β‐gal (Figure 2H), we found that the concentration of 1 μmol/L had a better intervention effect. Consistently, CO (1 μmol/L) also decreased the positive expression of SA‐β‐gal in HK‐2 and HPC (Figure 2I). Taken together, these findings unravelled that CO played remarkable anti‐senescence role both in vivo and in vitro.

### 3.3 CO activated autophagy and improved autophagy flow in vivo and in vitro

**FIGURE 3:**
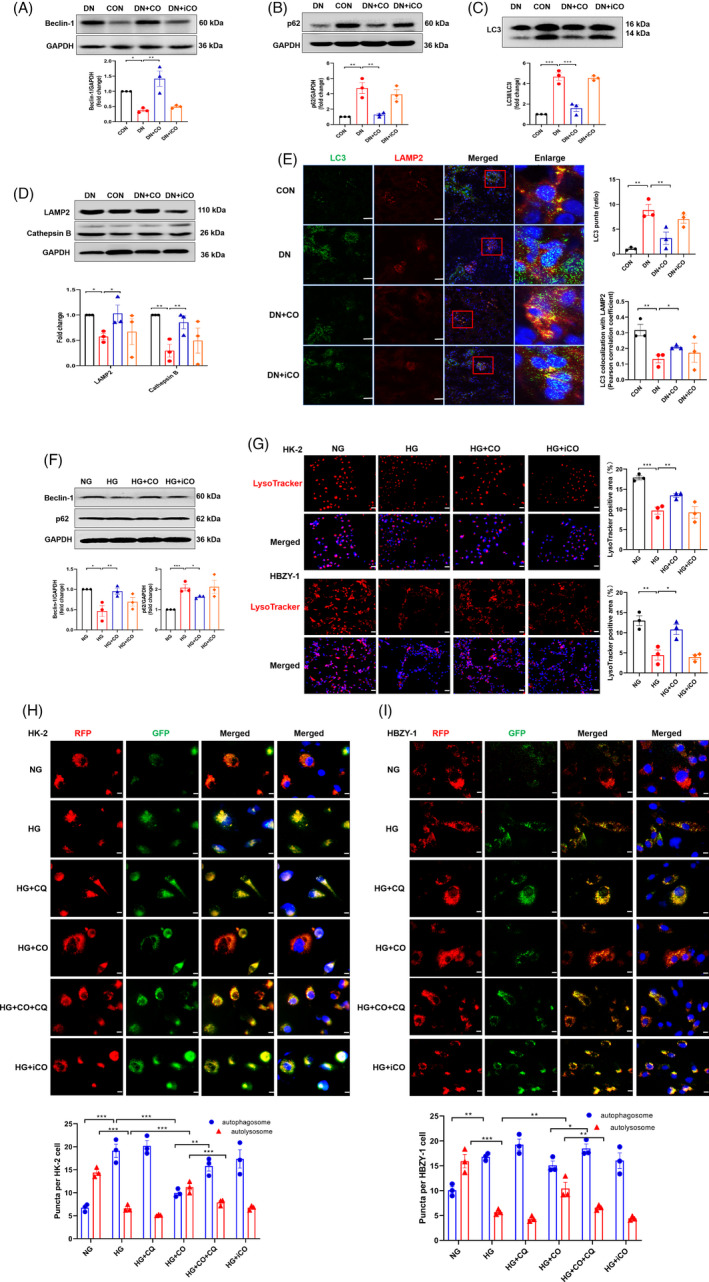
CO activated autophagy and improved autophagy flow in DN mice. A, B, C, Western blot was performed to measure expression of autophagy‐related proteins including autophagy initiating protein Beclin‐1, autophagy substrate p62 and autophagosome marker LC3 (n = 3). D, Immunoblots of renal cell lysate and densitometric analysis of the markers of lysosomal function (LAMP2 and Cathepsin B) (n = 3). E, Representative confocal microscopy images of LC3 puncta and LAMP2 co‐localization of renal tissue (Scale bar: 50 μm) and quantification of LC3 puncta and co‐localization between LC3 puncta and LAMP2 (n = 3). F, Immunoblots and densitometric analysis of Beclin‐1 and p62 in HK‐2 (n = 3). G, Representative microscopy images and quantification of HK‐2 and HBZY‐1 incubated with LysoTracker (100 nmol/L) (n = 3). Scale bar: 50 μm. H,I, Representative microscopy images of RFP‐GFP‐LC3 in HK‐2 and HBZY‐1 (Scale bar: 10 μm). Statistical results of autophagosomes and autolysosomes puncta in different groups (CORM‐2: 1 μm and CQ: 5 μmol/L) (n = 3). *P < .05, **P < .01, ***P < .001

A growing body of research revealed the negative regulation of autophagy to senescence, and CO has been considered as an autophagy activator. To explore the effects of CO on autophagy in DN, a series of autophagy‐related proteins were measured. Compared with diabetic mice, CO treatment significantly increased the expression of autophagy initiation protein Beclin‐1 (Figure 3A) and decreased the autophagy substrate p62 (Figure 3B), but reduced the ratio of LC3Ⅱ to LC3Ⅰ, the marker of autophagosome (Figure 3C). Previous evidence suggested that this contradiction may be due to blocked autophagy flow caused by dysfunction of lysosome.^25^ Results showed that lysosome‐related proteins Cathepsin B and LAMP2 were increased in the DN + CO group compared to DN group (Figure 3D). Moreover, the co‐localization analysis of LC3 puncta and LAMP2 supported that CO markedly reduced the accumulation of autophagosomes and increased the number of autolysosomes (Figure 3E).

Consistently, the dysfunction of autophagy and lysosome was found in vitro, as shown by protein levels and lysosomal probes (Figure 3F,G). Further, HK‐2 and HBZY‐1 were transfected with lentivirus RFP‐GFP‐LC3 to monitor autophagy flow. Under the condition of HG, puncta of autophagosomes were increased and autolysosomes were decreased in HBZY‐1 and HK‐2, which was not further changed with chloroquine (CQ) treatment. CO increased the number of autolysosomes and decreased autophagosomes, which was inhibited by the addition of CQ (Figure 3H,I). In summary, these results demonstrated that CO activated autophagy and improved autophagy flow in vivo and in vitro.

### 3.4 CO alleviated senescence through improving autophagy in vitro

**FIGURE 4:**
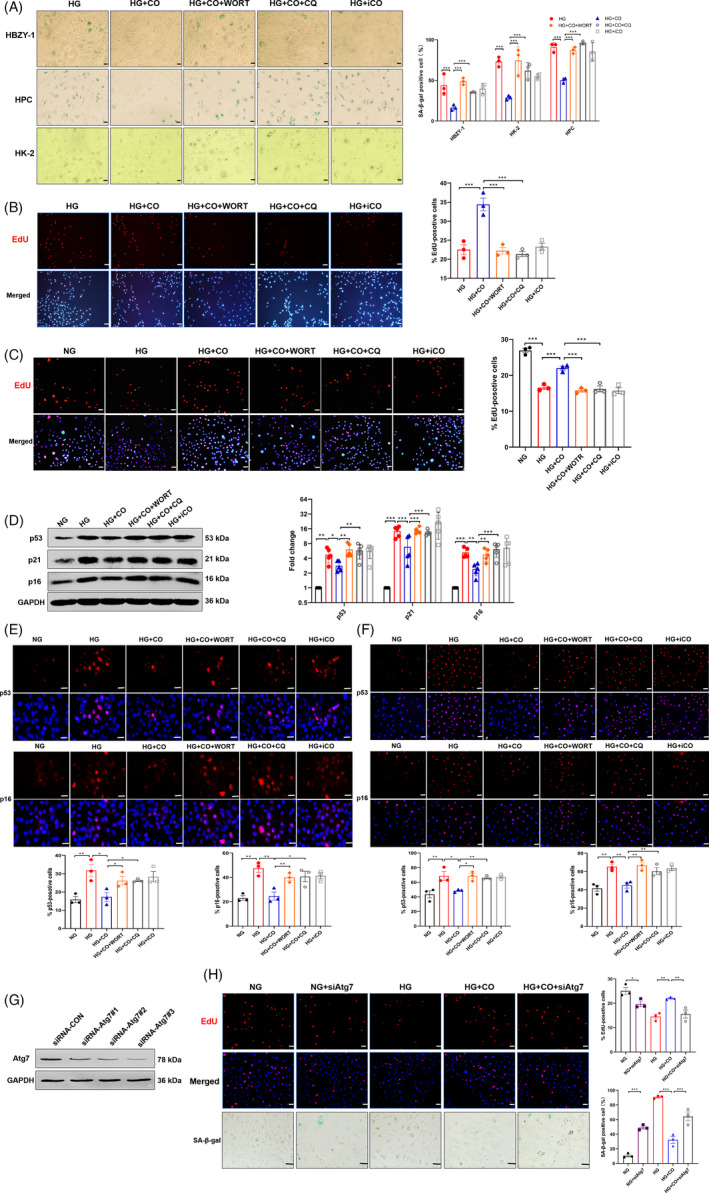
CO alleviated senescence through improving autophagy in vitro. HBZY‐1, HPC and HK‐2 were treated with HG, CORM‐2 and autophagy inhibitors (WORT: 200nM and CQ: 5 μmol/L). A, The staining and positive cells of SA‐β‐gal in 3 kinds of renal cells (n = 3). Scale bar: 25 μm. B,C, Cell proliferation assessed by EdU staining in HBZY‐1 and HK‐2 and quantification of the proportion of positive cells (n = 3). Scale bar: 50 μm. D, Expression of senescence‐related protein (p53, p16 and p21) in HBZY‐1 (n = 5). E,F, Representative immunofluorescence images of p53 and p16 and quantification of the proportion of positive cells in HK‐2 and HPC (n = 3). Scale bar: 50 μm. G, The protein expression levels of Atg7 in HK‐2 silenced with siRNA (three sequences). H, Representative images of SA‐β‐gal (Scale bar: 50 μm) and EdU staining (Scale bar: 25 μm), and quantification of the proportion of positive cells in HK‐2 (n = 3). *P < .05, **P < .01, ***P < .001

To further explore the role of autophagy in the anti‐senescence mechanism of CO, a combination of CORM‐2 and autophagy inhibitors was used in renal cells exposed to HG. Two types of inhibitors, wortmannin (WORT) to block autophagy initiation and CQ to restrain autophagy flow, effectively reversed the reduction of SA‐β‐gal by CO in 3 types of renal cells (Figure 4A). Consistently, CO significantly increased the proportion of EdU^+^ cells, which was inhibited by two inhibitors in HBZY‐1 (Figure 4B) and HK‐2 (Figure 4C). In HBZY‐1, the senescence‐alleviated effect of CO, shown by the reduced expression of p53, p21 and p16, was absent after the addition of autophagy inhibitors (Figure 4D). Similarly, immunofluorescence analysis showed that autophagy inhibitors blocked the decreases of p53‐ and p16‐positive cells by CO in HK‐2 (Figure 4E) and HPC (Figure 4F). Further, the silence of Atg7 (Figure 4G) blocked the protective effects of CO, shown by increased SA‐β‐gal^+^ cells and decreased EdU^+^ cells in HK‐2 (Figure 4H). The aforementioned results suggested that CO suppressed senescence by improving autophagy.

### 3.5 Activated autophagy may degrade SASP in DN mice

**FIGURE 5:**
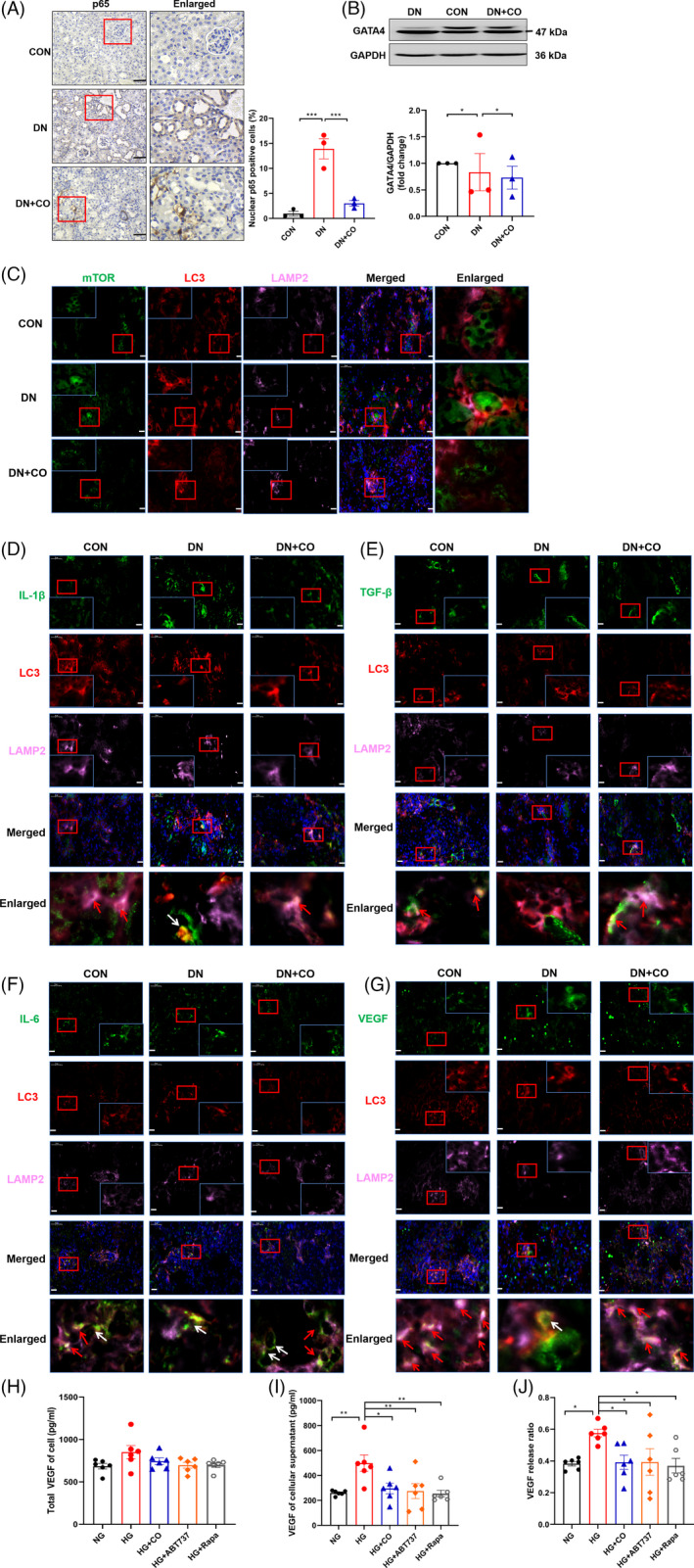
Activated autophagy may degrade SASP in DN mice. A, Immunohistochemical images and quantification of p65 expression and nuclear translocation (n = 3). Scale bar: 50 μm. B, Western blot analysis of GATA4 (n = 3). C, Representative immunofluorescence images of mTOR, LC3 puncta and LAMP2 co‐localization in kidney. Scale bar: 25 μm. D‐G, Representative immunofluorescence images showed the triple co‐localization of IL‐1β, IL‐6, TGF‐β and VEGF with LC3 and LAMP2 (the red arrows) and the white arrows indicated the location with LC3. Scale bar: 25 μm. H, The total VEGF protein level (cell lysate and supernatant) in HPC treated with different formulations (HG, ABT737: 1 mmol/L, rapamycin: 10 nmol/L, and CORM‐2). I, VEGF protein expression in the supernatant of HPC treated differently. J, VEGF protein release ratio of HPC (supernatant/total lysate) (n = 6). *P < .05, **P < .01, ***P < .001

We further explored the specific relationship between autophagy and senescence. Research showed that autophagy alleviated senescence by selectively degrading GATA4, which positively regulated NF‐κB to release SASP.^26^ In line with this, a large amount of SASP was developed in the DN group (Figure 2D), and the expression and nuclear translocation of p65 were increased (Figure 5A). However, the protein level of GATA4 stayed unchanged, suggesting GATA4 may not mediate the generation of SASP in high glucose state (Figure 5B). Additionally, studies revealed that TOR‐autophagy spatial coupling compartment (TASCC) formed by the combination of mTOR, late autophagosome and lysosome accelerated senescence by increasing the secretion of SASP.^27^ However, immunofluorescence results showed that there was no triple co‐localization of mTOR, LC3 and LAMP2, indicating the failed formation of TASCC in the kidneys (Figure 5C).

As we know, autophagy functions in the degradation of cytosolic constituents, which is mainly performed by autolysosome formed by the fusion of autophagosome and lysosome. Autophagy has been reported to reduce the secretion of IL‐1β by degrading pro‐IL‐1β in LPS‐stimulated macrophage.^28^ Interestingly, we found that IL‐1β (green) was co‐located with LC3 puncta (red) and LAMP2 (pink) in the CON and DN + CO groups (Figure 5D, the red arrow), indicating the intersection between SASP and autophagy degradation. Similarly, in comparison with the DN group, other cytokines of SASP including IL‐6, TGF‐β and VEGF also had modest but evident co‐localization with LC3 puncta and LAMP2 in the CON and DN + CO groups (Figure 5E‐G, the red arrow), among which VEGF co‐localized with autolysosomes most obviously (Figure 5G). However, the overlapping yellow staining in the DN group showed SASP was only co‐located with LC3 puncta (Figure 5D‐G, the white arrow). Furthermore, the protein level of VEGF was measured in HPC after adding autophagy agonists (ABT737 and rapamycin) or CORM‐2. Although there was no significant difference in total protein level of VEGF (Figure 5H), the presence of both autophagy agonists and CO markedly reduced the release of VEGF in HPC induced by HG (Figure 5I,J). Taken together, these results indicated that some SASP is degraded via autophagy.

### 3.6 CO activated autophagy partly by dissociating Beclin‐1‐Bcl‐2 complex

**FIGURE 6:**
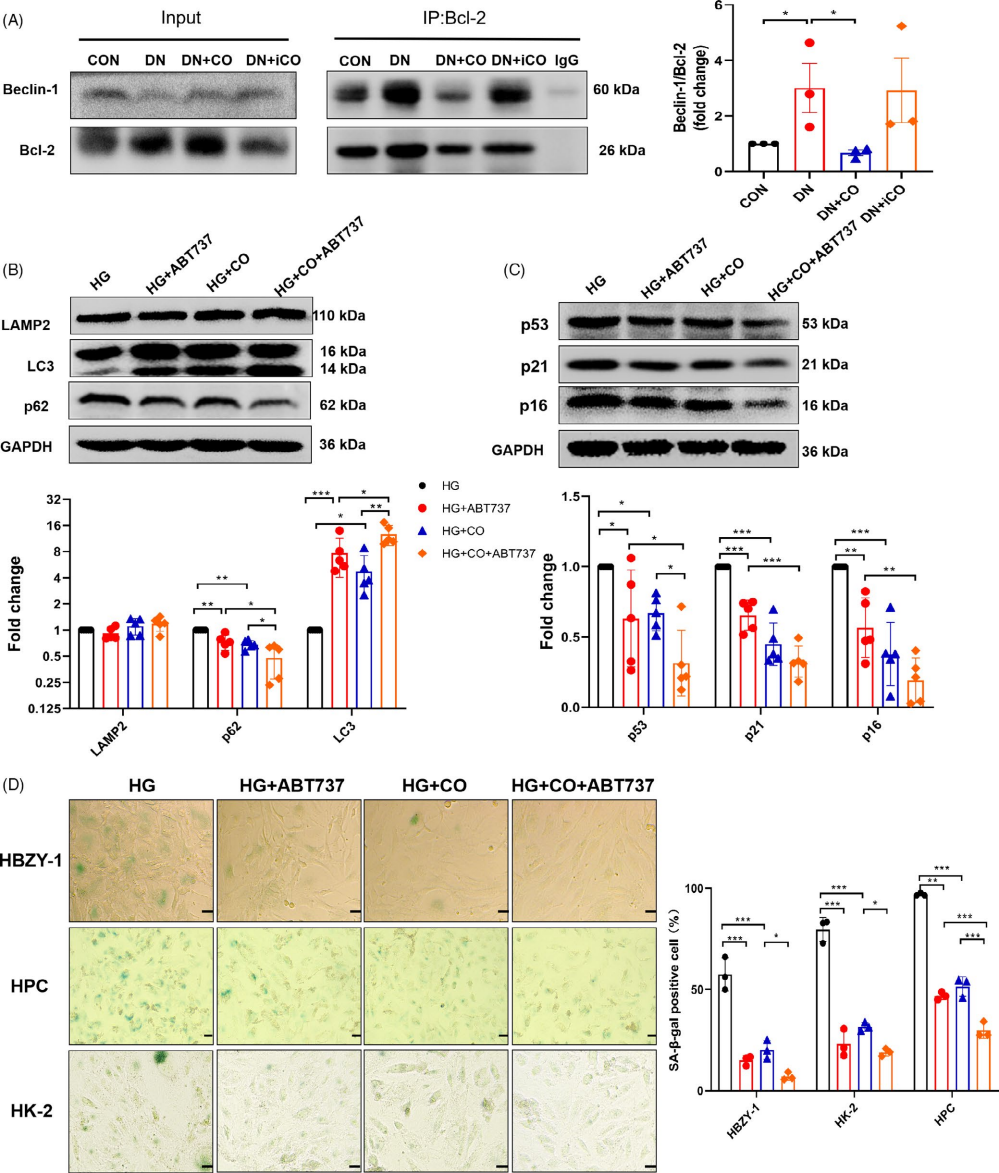
CO activated autophagy partly by dissociating Beclin‐1‐Bcl‐2 complex. A, Co‐immunoprecipitation of Beclin‐1 and Bcl‐2 and quantification of the ratio of Beclin‐1 to Bcl‐2 in the kidney (n = 3). B, Western blot analysis of a series of autophagy proteins (LAPM2, p62 and LC3) in HBZY‐1 treated differently (HG, CORM‐2 and Beclin‐1‐Bcl2 complex dissociation agent ABT737) (n = 5). C, Quantification of the expression of p53, p21 and p16 (n = 5 or 6). D, The SA‐β‐gal staining images of 3 kinds of renal cells treated differently and statistical results (n = 3). Scale bar: 50 μm. *P < .05, **P < .01, ***P < .001

The dissociation of Beclin‐1‐Bcl‐2 complex has been reported to improve autophagy as well as prevent premature ageing including age‐related renal changes.^21^ Additionally, CO activated autophagy through Beclin‐1 in the sepsis mice.^17^ Hence, we explored whether dissociating the complex mediated the activation of autophagy by CO. Co‐immunoprecipitation results showed that CO reduced Beclin‐1‐Bcl‐2 binding in DN mice (Figure 6A). Furthermore, compared with the treatment of HG and CO, the combined use of HG, CO and the Beclin‐1‐Bcl‐2 complex dissociation agent ABT737 further activated autophagy of HBZY‐1, as evidenced by unchanged LAMP2, decreased p62 and increased LC3Ⅱ/LC3Ⅰ ratio (Figure 6B). Moreover, the results showed that the combined use of CO and ABT737 further down‐regulated the expression of p53, p21 and p16 in HBZY‐1 (Figure 6C) and decreased the SA‐β‐gal^+^ cell in HBZY‐1, HK‐2 and HPC (Figure 6D), indicating that ABT737 enhanced the anti‐senescence effect of CO. These observations suggested that CO could activate autophagy by dissociating Beclin‐1‐Bcl‐2 complex to alleviate senescence.

## 4 DISCUSSION

Accumulating senescent cells are increasingly considered to be malignant ‘zombie cells’ resistant to death, with the capacity of inducing tissue dysfunction and fueling age‐related diseases. Researchers reported that transplantation of senescent precursor fat cells into 6‐month‐old mice caused a dose‐dependent decline in body function.^29^ Conversely, removal of senescent cells improved a range of age‐related diseases such as T2D,^30^ osteoarthritis^31^ and Alzheimer's disease.^32^ Our results displayed the occurrence of senescence in DN mice and 3 types of renal cells exposed to HG. These results and previous research consistently suggested the presence of senescence in DN. Interestingly, among the 9 kinds of tissue of 27‐month‐old SD rats, renal senescence was the earliest and most serious, indicating kidney was susceptible to senescence.^33^ Based on these findings, we speculated that the therapies targeting senescent cells may protect against DN. Recent limited evidence showed that CO alleviated drug‐induced senescence of endothelial cell. Similarly, we provided results that CO significantly down‐regulated a great deal of senescent phenotype and accordingly recovered renal function in DN mice. While CO has been proven to exert renoprotection against ischaemia‐reperfusion and kidney transplantation mice via anti‐inflammatory, anti‐apoptosis and other classic pathways,^34^ our study proposed a novel mechanism that CO confers improvement from renal senescence of DN mice.

Except the widely recognized p53/p21 and p16 pathways, senescence has been described to be regulated by autophagy positively.These results and previous research consistently suggested the presence of senescence in DN Consistent with this, the reduction of autophagy was found in kidney with ageing.^35^ Moreover, there was a negative correlation between Atg5 and renal ageing of DN mice, suggesting the potentiality of anti‐senescence of autophagy in DN. Our results showed that the dysfunction of autophagy and autophagy flow in vivo and vitro were positively associated with accumulated senescent cells. Interestingly, CO has been considered to be an effective autophagy activator. In our study, CO activated autophagy, but decreased autophagosomes. The further data displayed that CO ameliorated lysosomal function and autophagy flow by the transfection of RFP‐GFP‐LC3. Furthermore, autophagy inhibitors and siAtg7 restrained the improvement of senescence by CO in vitro, supporting that CO slowed senescence via autophagy.

Not only that, the dissociation of Beclin‐1‐Bcl‐2 complex by CO was observed in DN mice. To our knowledge, Beclin‐1 functions as autophagy initiator when dissociated from Bcl‐2. The disruption of the complex may be a potential target for autophagy activated by CO. In addition, knocking out the Beclin‐1 and Bcl‐2 binding sites not only activated autophagy but improved ageing and the lifespan of mice,^21^ suggesting the complex coordinately regulated autophagy and senescence. Our experiments showed that this complex dissociation agent ABT737 further enhanced the activation of autophagy and improvement of senescence by CO in vitro. Consistent with our results, disrupting interaction of Beclin‐1‐Bcl‐2 complex positively influence multiple disease, including protecting against cardiac injuries of diabetic cardiomyopathy mice^36^ and exerting renoprotection on mice challenged by ischaemia‐reperfusion.^37^ These observations indicated that CO may activate autophagy by dissociating Beclin‐1‐Bcl‐2 complex to relieve senescence and renal injuries.

What's more, the specific relationship between autophagy and senescence requires further elucidation. Autophagy is a cleansing process to deliver enclosed constituents to lysosome for degradation. Researchers evidenced that autophagy reduced the secretion of IL‐1β, a major SASP component, through the degradative pathway.^38^ Surprisingly, the co‐localization of IL‐1β, LC3 puncta and LAMP2 was observed in kidneys, suggesting the degradation of IL‐1β by CO treatment in DN mice. Besides, intracellular IL‐6, TGF‐β and VEGF, respectively, co‐localized with LC3 puncta and LAMP2, indicating the intersection between autophagy and the degradation of SASP. Consistently, the drug‐activated autophagy was proven to reduce the secretion of IL‐6 in chondrocytes,^39^ while the suppression of autophagy by deleting Atg7 caused increased export of IL‐1β, IL‐6 and IL‐8 in adipocytes and breast cancer cells.^40^ However, IL‐1α was reported not to be a direct substrate for autophagic elimination in macrophages,^41^ suggesting that SASP was partly degraded by autophagy.

Noteworthily, we observed that SASP co‐located with autophagosomes in the kidney of DN mice (Figure 5D‐G, the white arrow), indicating SASP may be engulfed by autophagosomes but escape lysosomes. This is potentially attributed to secretory autophagy that autophagosomes are not delivered to lysosomes but instead fuse directly with plasma membrane, leading to the release of cytosolic components (SASP) to the extracellular space.^42^ However, the co‐localization of SASP and autolysosomes increased greatly in normal and CO‐intervened mice (Figure 5D‐G, the red arrow). The possible explanation for this phenomenon is that both autophagy‐mediated secretion and degradation participate in SASP transportation.^43^ Although the regulation of the divergence between excretion versus degradation of the intracellular contents remains to be fully understood, we found both CO and autophagy agonists blocked the export of VEGF in HPC treated with HG, suggesting activated autophagy contributed to SASP degradation. Additionally, impaired lysosomal function was reported to cause the release of autolysosome materials in microglial cell.^44^ Thus, CO‐induced activation of autophagy and restoration of lysosomal function may lead to more degradation of SASP than secretion.

Much evidence demonstrated that secreted SASP played a negative role in some diseases. For example, the SASP was reported to facilitate retinal vascular damage by transmitting senescence in retinopathy mice,^45^ induce senescence and injure of non‐senescent biliary cells.^46^ However, it is unknown whether SASP is responsible for DN progression. Omics results showed that inflammatory cytokines were up‐regulated in early and especially in late DN.^47^ Consistently, we found lots of SASP was developed in DN mice, among which IL‐1β was increased most significantly compared to normal mice and VEGF was co‐localized with autolysosomes most obviously in normal and CO‐intervened mice. And the treatment of anti‐IL‐β IgG attenuated kidney injuries including fibrosis in db/db mice.^48^ The deletion of VEGF‐B protected against DN by reducing renal lipid accumulation,^49^ while the overexpression of VEGF‐A in adult mice exacerbated renal dysfunction and structure change.^50^ The above data on the regulation of SASP further suggested the key role of SASP in the DN. Our study also showed that the administration of CO inhibited the expression and secrete of SASP, subsequently improving renal fibrosis and structural changes. Hence, we inferred that the blockage of SASP‐mediated senescence through degradation pathway of autophagy may be one of the mechanisms by which CO protects HFD + STZ‐induced DN.

Taken together, the present findings indicated that CO alleviated renal senescence of DN through the improvement of autophagy mediated by dissociating Beclin‐1‐Bcl‐2 complex, which was partly attributed to the degradation of SASP. Our study sheds new light on the prevention and treatment of DN and regulation of renal senescence. Expectantly, the specific effects of senescence on DN are worthy of further investigation. On the other hand, anti‐senescence drugs may become a breakthrough in the treatment of DN and other age‐related diseases.

## CONFLICT OF INTEREST

The authors have no conflicts of interest to declare.

## AUTHOR CONTRIBUTIONS

All authors contributed to the study. The study was designed by Yuhan Tang, Chao Gao and Ping Yao. Material preparation was performed by Chunjie Jiang and Dan Li. The animal model was guided by Guibin Mei and Ying Zhao. The experiments were performed by Li Chen, Xueer Cheng, Huimin Chen and Cheng Wan. The manuscript was written and polished by Yuhan Tang, Li Chen and Guibin Mei. All authors read and approved the final manuscript.

## Supporting information

Supplementary Material

## Data Availability

The data, analytical methods and study materials will be made available to other researchers for purposes of replicating the procedure and are available by contacting the corresponding authors.
