# Supplementary material for: Carbon monoxide alleviates senescence in diabetic nephropathy by improving autophagy
Source: Cell Prolif. 2021 May 7;54(6):e13052. doi: 10.1111/cpr.13052 (PMC8168421; doi:10.1111/cpr.13052)
Supplement: Supplementary file 1 — Supplementary Material [file CPR-54-e13052-s001.pptx]

## Slide 1
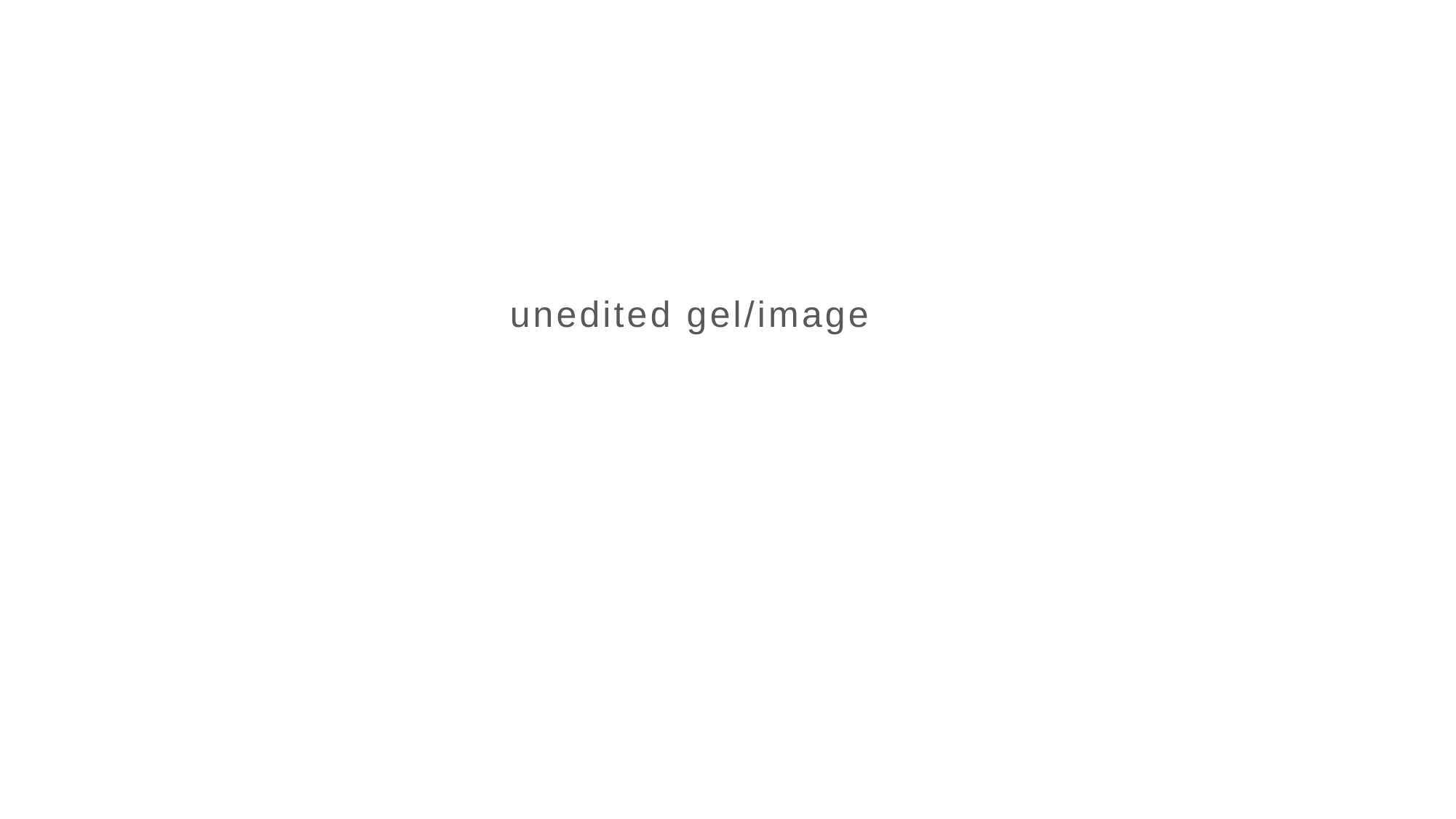

unedited gel/image

## Slide 2
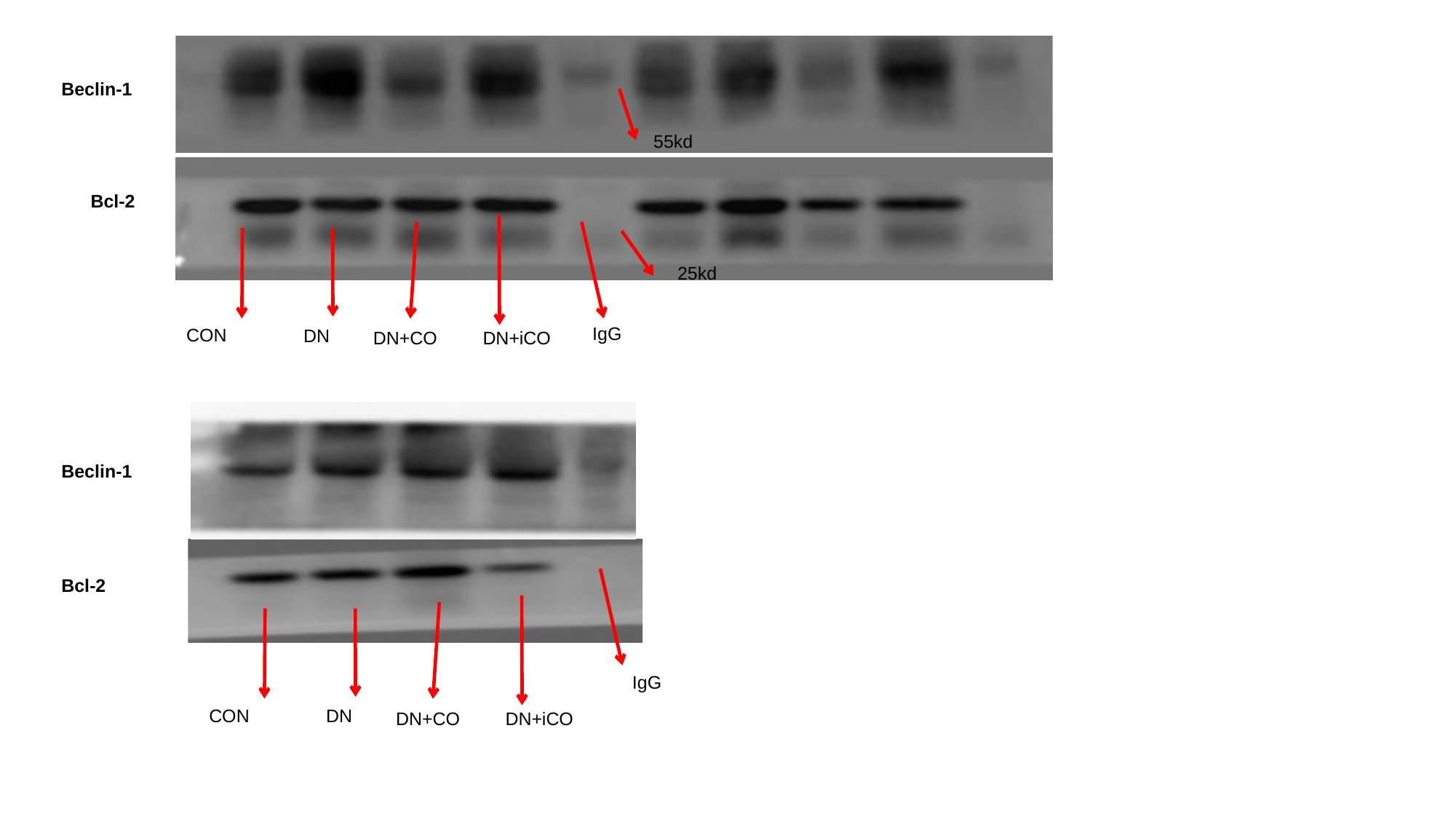

Beclin-1
55kd
Bcl-2
25kd
IgG
CON
DN
DN+CO
DN+iCO
Beclin-1
Bcl-2
IgG
CON
DN
DN+CO
DN+iCO

## Slide 3
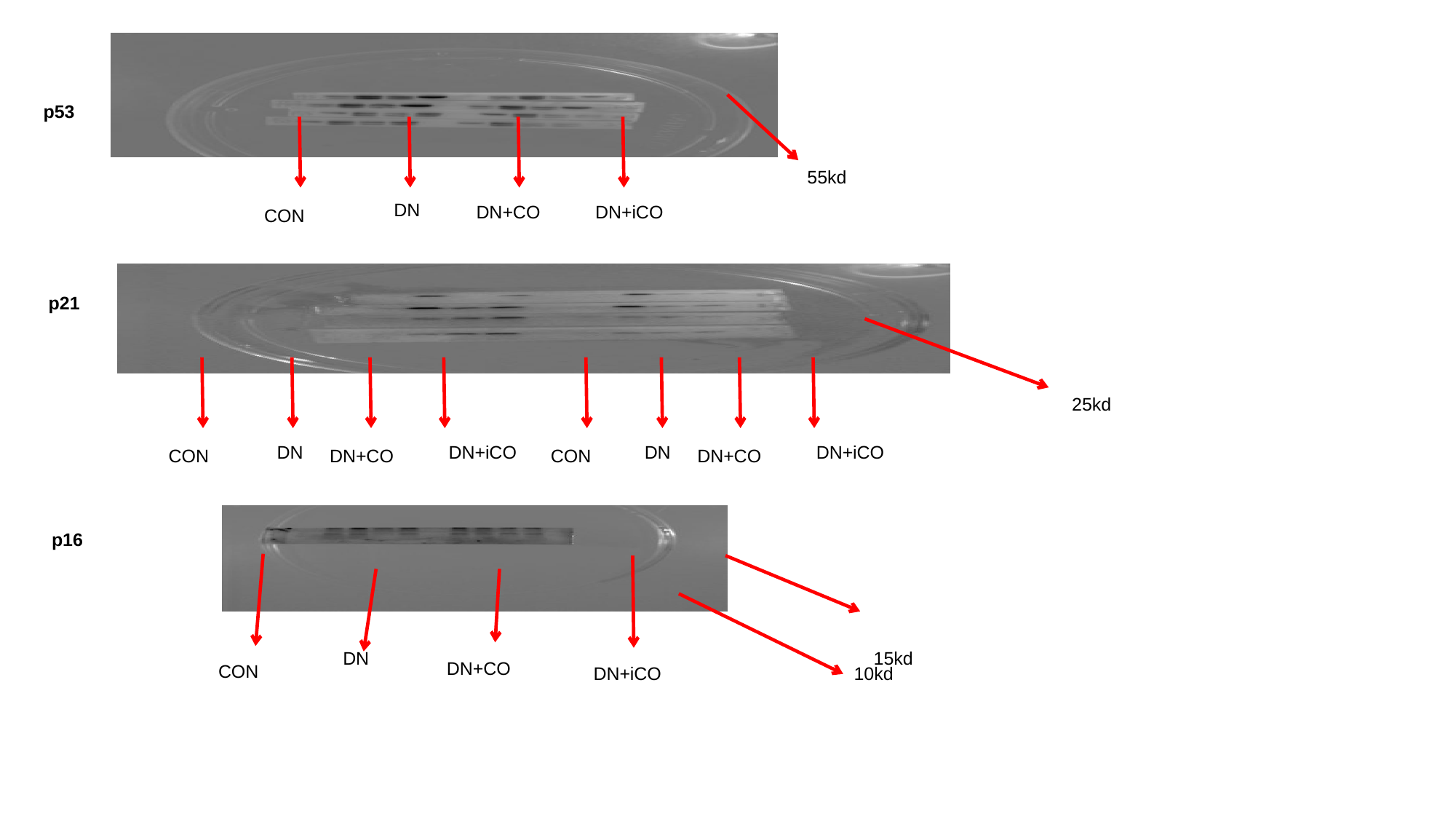

p53
55kd
DN
DN+CO
DN+iCO
CON
p21
25kd
DN
DN+iCO
DN
DN+iCO
CON
DN+CO
CON
DN+CO
p16
DN
15kd
DN+CO
CON
DN+iCO
10kd

## Slide 4
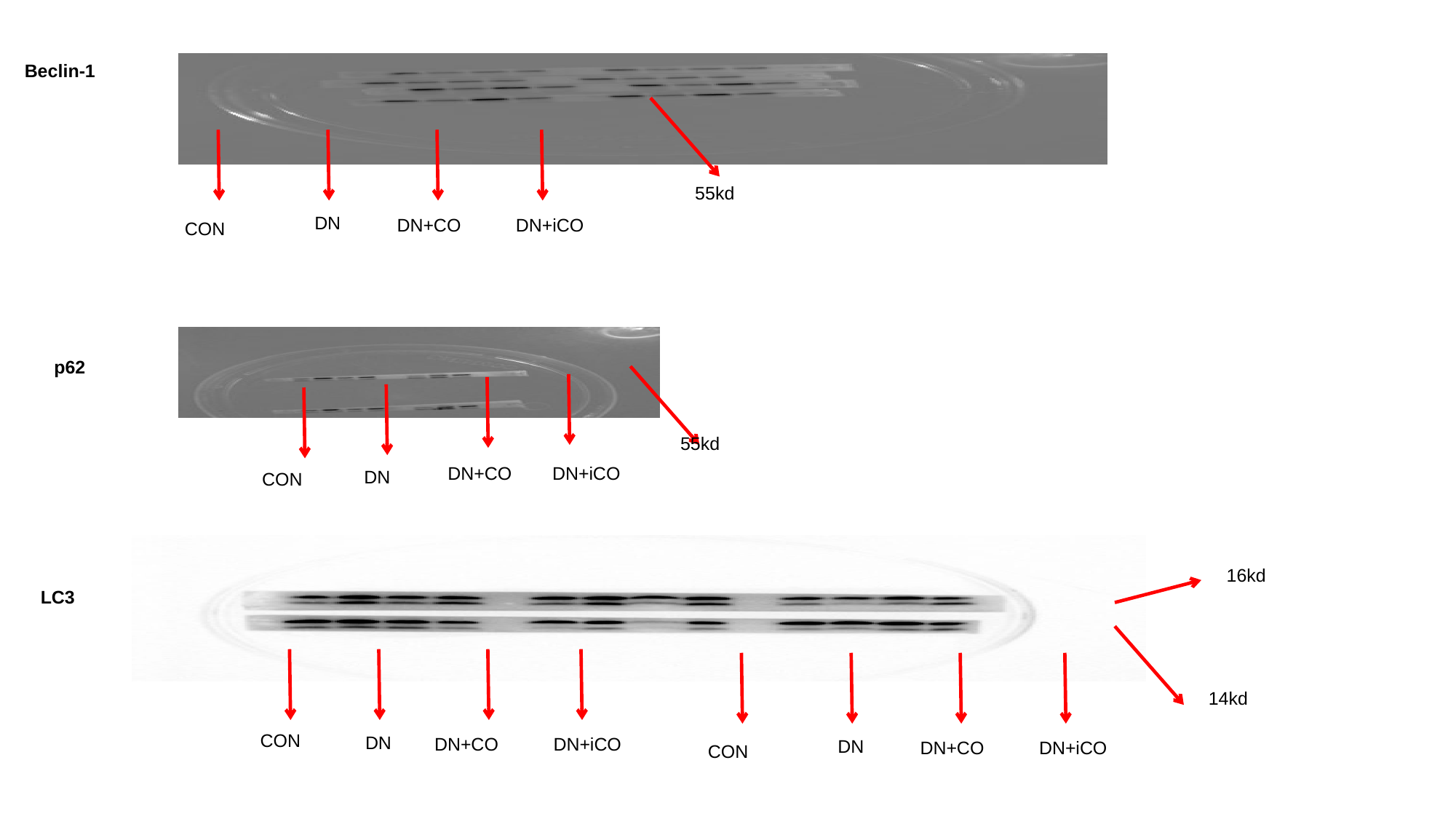

Beclin-1
55kd
DN
DN+CO
DN+iCO
CON
p62
55kd
DN+CO
DN+iCO
DN
CON
16kd
LC3
14kd
CON
DN
DN+CO
DN+iCO
DN
DN+CO
DN+iCO
CON

## Slide 5
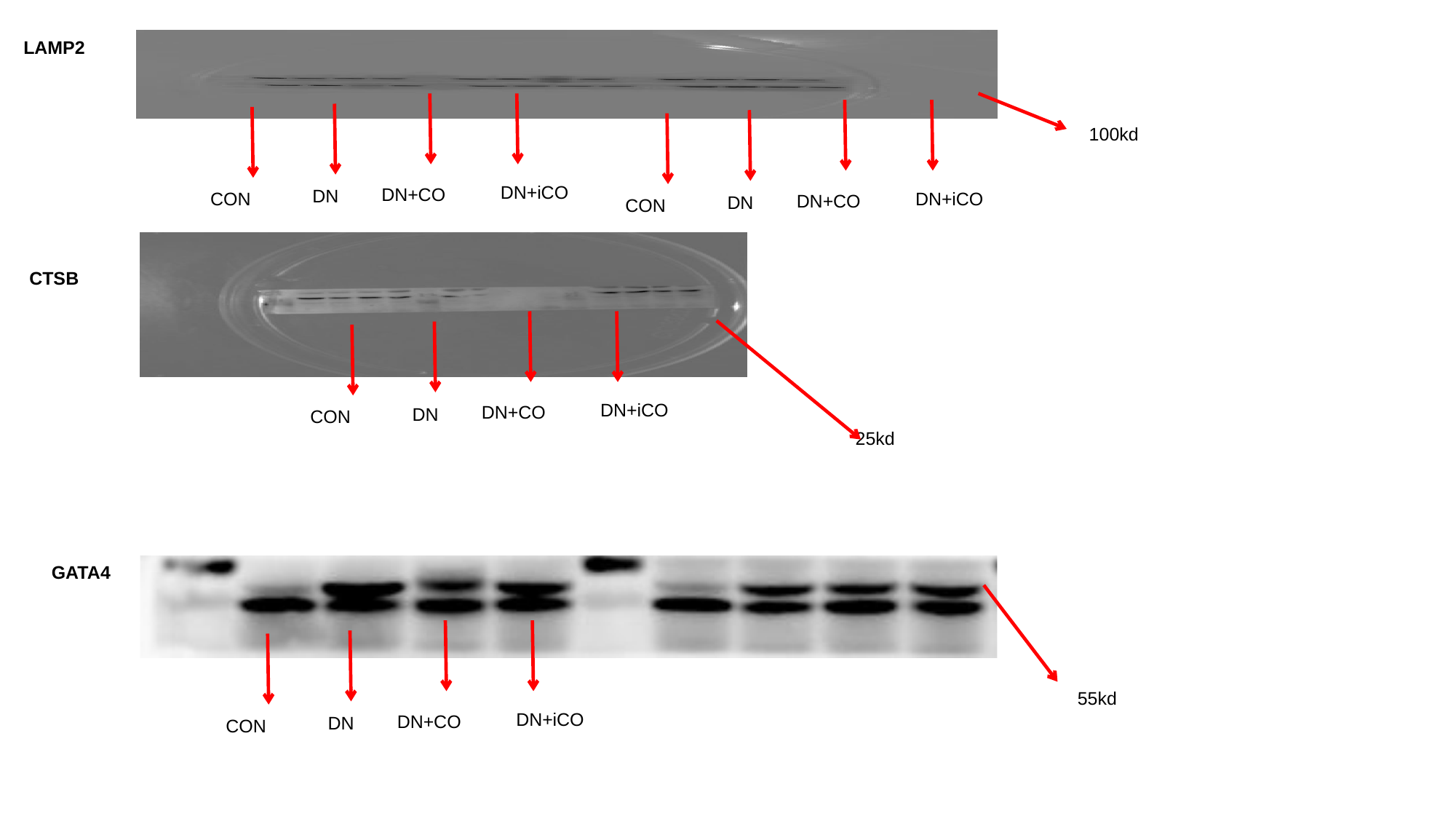

LAMP2
100kd
DN+iCO
DN+CO
DN
CON
DN+iCO
DN+CO
DN
CON
CTSB
DN+iCO
DN+CO
DN
CON
25kd
GATA4
55kd
DN+iCO
DN+CO
DN
CON

## Slide 6
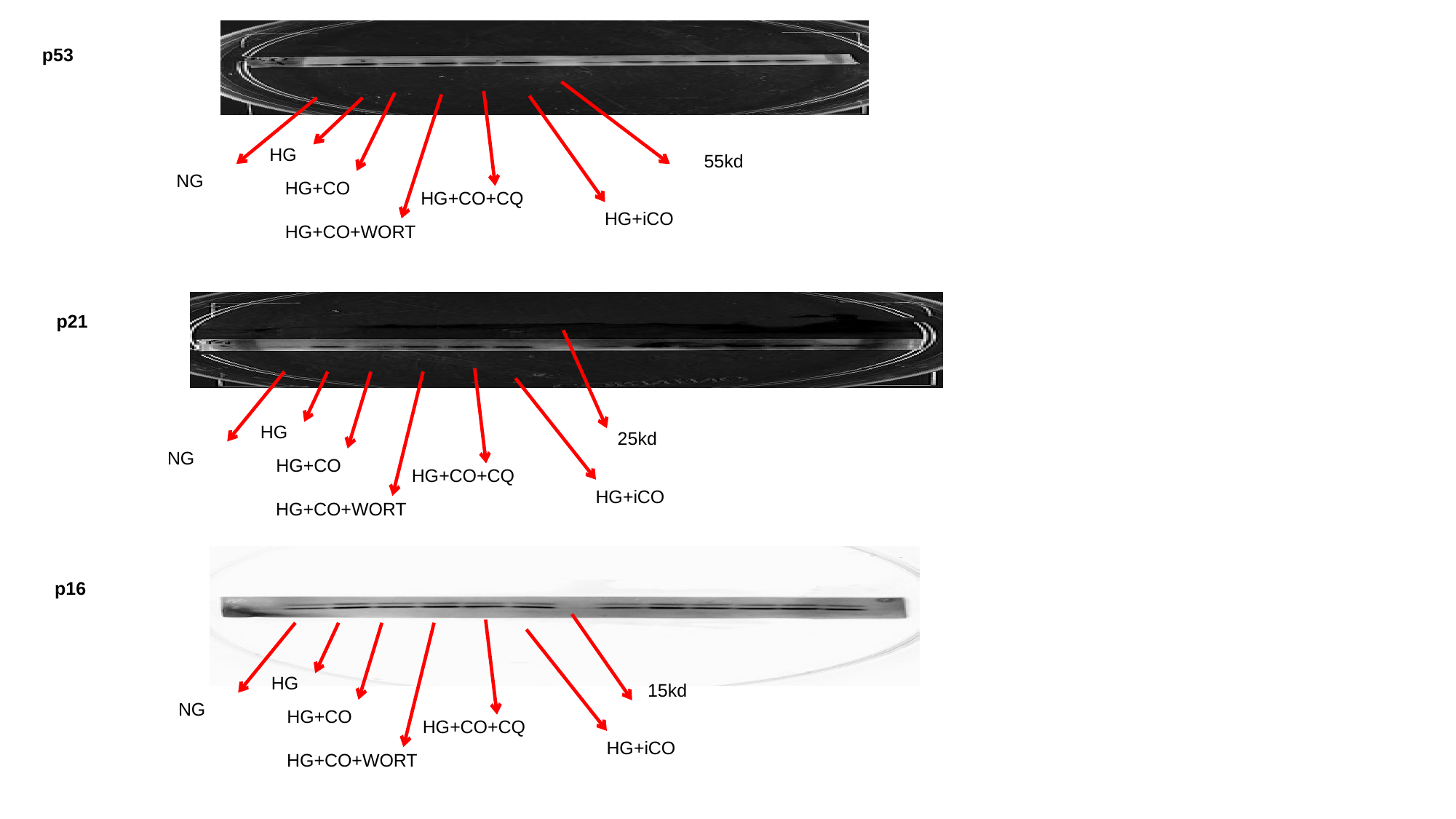

p53
HG
55kd
NG
HG+CO
HG+CO+CQ
HG+iCO
HG+CO+WORT
p21
HG
25kd
NG
HG+CO
HG+CO+CQ
HG+iCO
HG+CO+WORT
p16
HG
15kd
NG
HG+CO
HG+CO+CQ
HG+iCO
HG+CO+WORT

## Slide 7
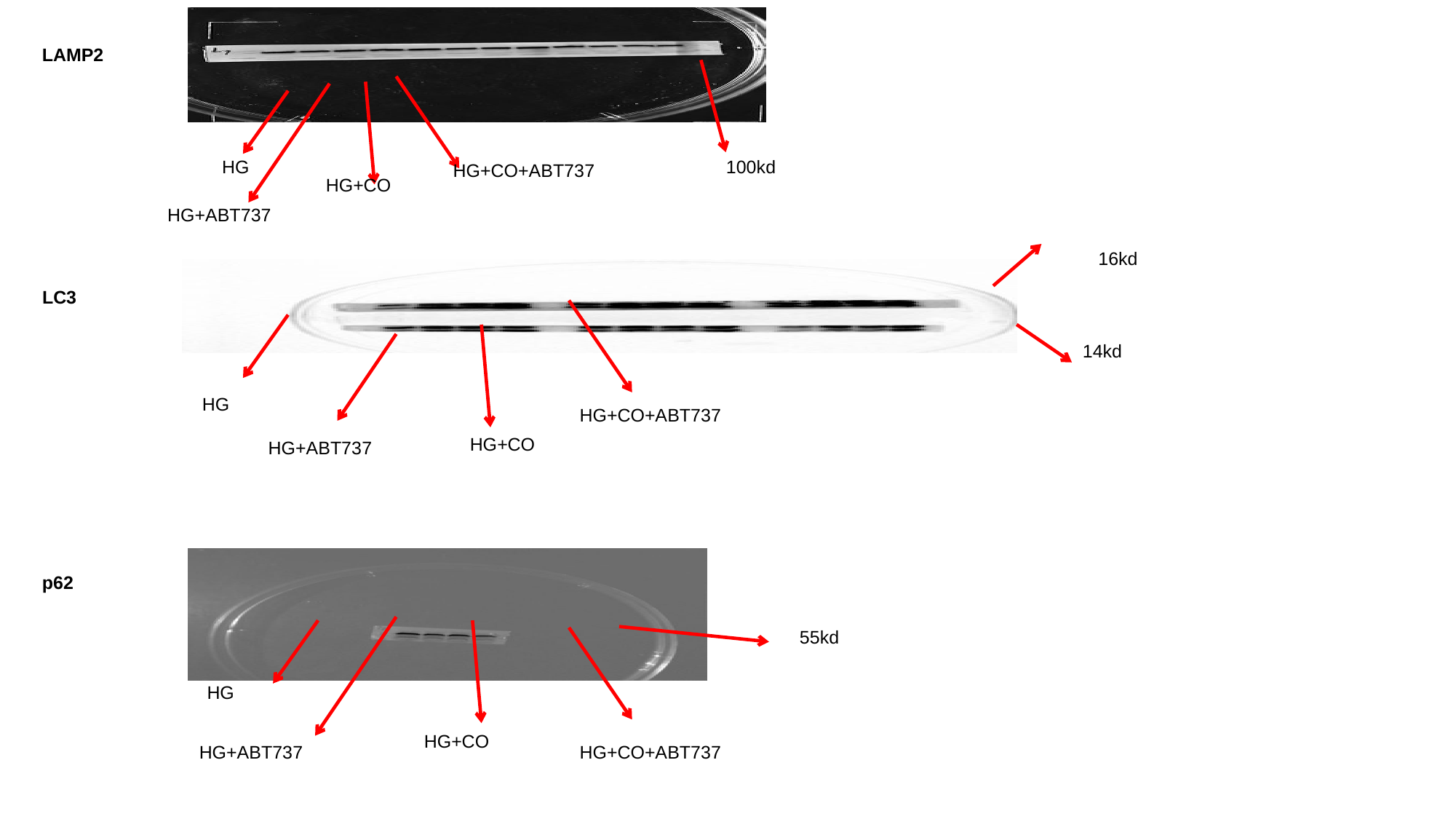

LAMP2
HG
100kd
HG+CO+ABT737
HG+CO
HG+ABT737
16kd
LC3
14kd
HG
HG+CO+ABT737
HG+CO
HG+ABT737
p62
55kd
HG
HG+CO
HG+ABT737
HG+CO+ABT737

## Slide 8
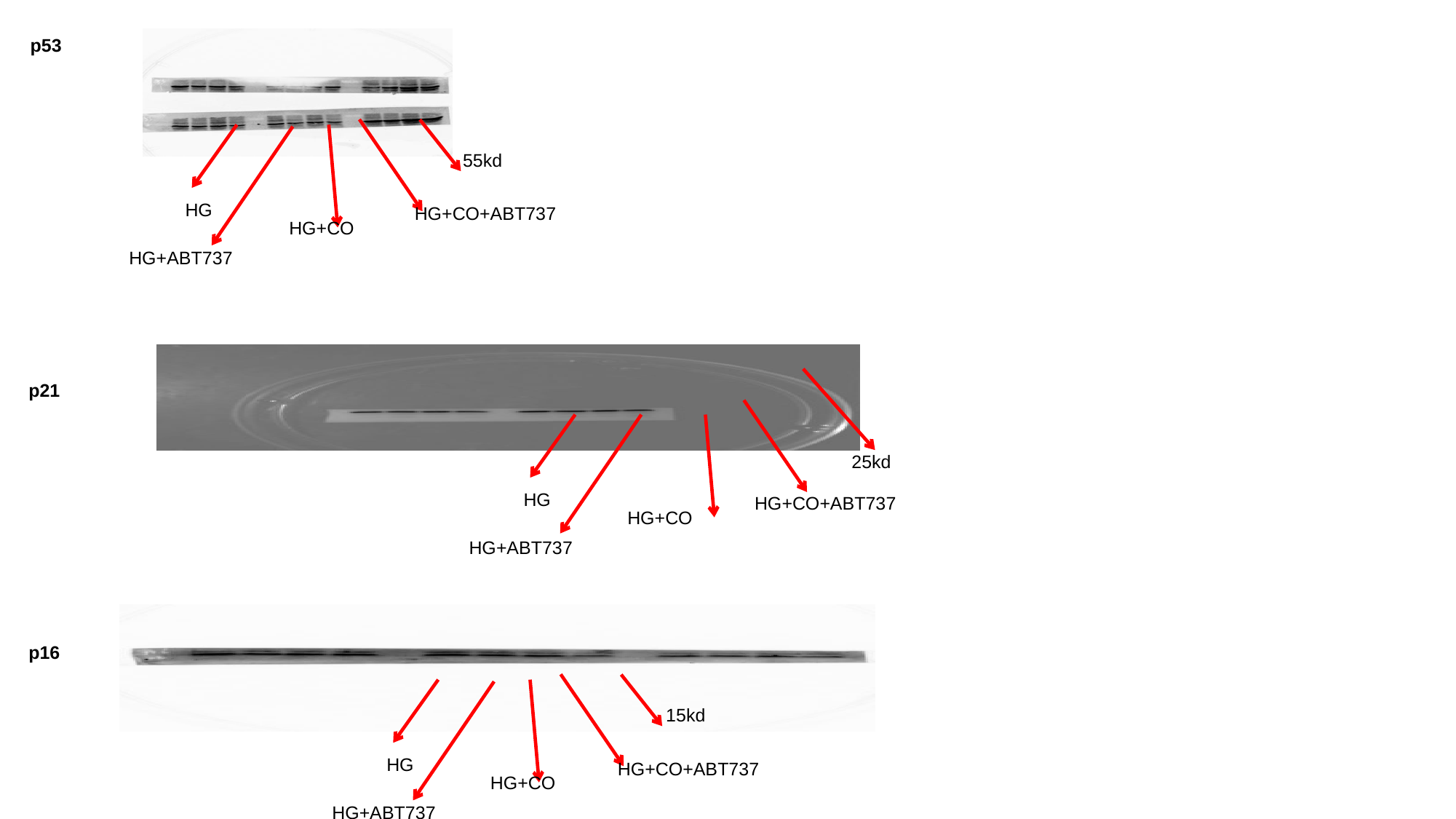

p53
55kd
HG
HG+CO+ABT737
HG+CO
HG+ABT737
p21
25kd
HG
HG+CO+ABT737
HG+CO
HG+ABT737
p16
15kd
HG
HG+CO+ABT737
HG+CO
HG+ABT737
